# Teledentistry support in COVID-19 oral care

**DOI:** 10.6061/clinics/2020/e2030

**Published:** 2020-06-08

**Authors:** Gabriel de Toledo Telles-Araujo, Raquel D’Aquino Garcia Caminha, Monira Samaan Kallás, Paulo Sergio da Silva Santos

**Affiliations:** Departamento de Cirurgia, Estomatologia, Patologia e Radiologia, Faculdade de Odontologia de Bauru, Universidade de Sao Paulo, Bauru, SP, BR

Given the latest updates in scientific publications about the impact of COVID-19 on oral health, such as the role of salivary glands as potential reservoirs for SARS-CoV-2 (1), the appearance of possible oral vesiculobullous lesions (2), and the presence maculopapular manifestations in suspected and confirmed cases (3), we aimed to emphasize the inexorable need for close monitoring by a dentist specialized in oral medicine in such COVID-19 patients. Their skill and accuracy in diagnosing mouth diseases could contribute to a better understanding of the pathogenesis of SARS-CoV-2 in oral health.

Based on the recent data, topical and systemic corticosteroid therapy is not recommended for COVID-19 infection. Hence, positive patients with immune-mediated oral conditions (pemphigus, pemphigoid, lichen planus) may present exacerbations of these manifestations during the period of viral infection. Correspondingly, multidrug therapies in patients positive for SARS-CoV2 and hospitalization conditions could also result in oral implications such as opportunistic infections, xerostomia, traumatic ulcerations due to orotracheal intubation, and periodontal disease (4).

Due to the pandemic scenario of COVID-19 in Brazil and following the recommendations of the Federal Council of Dentistry (5) and the National Health Surveillance Agency (6), dental care has been limited to emergencies and urgencies. Due to the aerosols produced during many oral procedures, this measure is justified because of the higher risk of exposure of theses professionals to the virus and prevention of cross-infection between patients. Unfortunately, this endorsement has created a distance between patients and dentists. Moreover, the incipient inclusion of a dentist in a hospital setting corroborates this detachment. Thus, there is still limited scientific evidence on the relationship between SARS-CoV-2 and oral diseases.

Aiming to bridge the gap between healthcare services and the population, telemedicine has been recognized and regulated in Brazil (7) as a tool to combat the pandemic of COVID-19. It favors the reduction of physical contact between professionals and patients and thus, spread of the virus. In dentistry, the following three modes of operation hold great value for oral diagnosis. Teleorientation allows professionals to perform screening, guide, and refer patients in isolation to face-to-face assistance, if needed. Telemonitoring permits professionals to visually monitor patients suspected or positive for SARS-CoV-2 who present oral lesions through photographic control. Finally, teleconsultation enables the exchange of information between professionals, assisting in the diagnosis and therapy to be instituted to the patient with greater agility and precision.

The systems available for executing telemedicine and/or teledentistry are still restricted, but represent an easy, viable, and accessible tool, useful for both health professionals and patients (8 9). This communication could occur through instant messaging applications (WhatsApp, Telegram, Instagram, SMS, Messenger) and video calling applications (Google Meet, Skype, Facetime, WhatsApp). A study published by Petruzzi et al. (9) confirmed the use of WhatsApp as a support in oral diagnosis, in which 82% of the teleconsulted cases agreed with the clinicopathological diagnosis, suggesting that it is a good option for teledentistry.

However, one limitation of this technology includes poor resolution of the images provided by the patient. The images must have an adequate resolution, without alterations with digital filters and must encompass the entire area of the mouth to be analyzed. These specifications will allow the correct evaluation by the oral medicine specialist. It is worth stressing that this diagnostic tool is palliative. Therefore, it is not possible to be used in all cases.

Thus, telemedicine is not a substitute for face-to-face consultation, and is mainly aimed at supporting the Brazilian Unified Public Health System (SUS) during the COVID-19 pandemic. Whenever physical consultation is required after teleorientation, the health measures of the regulatory agencies must be followed.
